# Whole exome sequencing of high-risk neuroblastoma identifies novel non-synonymous variants

**DOI:** 10.1371/journal.pone.0273280

**Published:** 2022-08-29

**Authors:** Weronika Przybyła, Kirsti Marie Gjersvoll Paulsen, Charitra Kumar Mishra, Ståle Nygård, Solveig Engebretsen, Ellen Ruud, Gunhild Trøen, Klaus Beiske, Lars Oliver Baumbusch

**Affiliations:** 1 Department of Pediatric Research, Division of Paediatric and Adolescent Medicine, Oslo University Hospital Rikshospitalet, Oslo, Norway; 2 Medical Faculty, Institute of Clinical Medicine, University of Oslo, Oslo, Norway; 3 Bioinformatics Core Facility, Institute for Cancer Research, Oslo University Hospital, Oslo, Norway; 4 ELIXIR-Norway, Institute of Informatics, University of Oslo, Oslo, Norway; 5 Norwegian Computing Center, Oslo, Norway; 6 Department of Paediatric Haematology and Oncology, Division of Paediatric and Adolescent Medicine, Oslo University Hospital Rikshospitalet, Oslo, Norway; 7 Department of Pathology, Oslo University Hospital Radiumhospitalet, Oslo, Norway; 2nd Medical Faculty Charles University Prague and Faculty Hospital Motol, CZECH REPUBLIC

## Abstract

Neuroblastoma (NBL), one of the main death-causing cancers in children, is known for its remarkable genetic heterogeneity and varied patient outcome spanning from spontaneous regression to widespread disease. Specific copy number variations and single gene rearrangements have been proven to be associated with biological behavior and prognosis; however, there is still an unmet need to enlarge the existing armamentarium of prognostic and therapeutic targets. We performed whole exome sequencing (WES) of samples from 18 primary tumors and six relapse samples originating from 18 NBL patients. Our cohort consists of 16 high-risk, one intermediate, and one very low risk patient. The obtained results confirmed known mutational hotspots in *ALK* and revealed other non-synonymous variants of NBL-related genes (*TP53*, *DMD*, *ROS*, *LMO3*, *PRUNE2*, *ERBB3*, and *PHOX2B*) and of genes cardinal for other cancers (*KRAS*, *PIK3CA*, and *FLT3*). Beyond, GOSeq analysis determined genes involved in biological adhesion, neurological cell-cell adhesion, JNK cascade, and immune response of cell surface signaling pathways. We were able to identify novel coding variants present in more than one patient in nine biologically relevant genes for NBL, including *TMEM14B*, *TTN*, *FLG*, *RHBG*, *SHROOM3*, *UTRN*, *HLA-DRB1*, *OR6C68*, and *XIRP2*. Our results may provide novel information about genes and signaling pathways relevant for the pathogenesis and clinical course in high-risk NBL.

## Introduction

Neuroblastoma (NBL) presents a major challenge in paediatric oncology due to its highly variable clinical appearance and extreme genetic heterogeneity [1–3]. It is the most common extra-cranial solid tumor in children, originating from immature precursors of sympathic ganglionic cells during embryonic and fetal life [4, 5]. The disease accounts for 7–8% of all childhood malignancies and 15% of all paediatric malignant deaths [6, 7].

NBL is diagnosed according to the histopathological criteria defined in the International Neuroblastoma Pathology Classification [8]. To assess the prognosis of individual patients, the International Neuroblastoma Risk Group (INRG) has developed a pre-treatment risk scheme built on clinical, pathological, and genetic factors like age, histologic category, grade of tumor differentiation, *MYCN* oncogene and chromosome 11q copy number variations (CNVs), and DNA ploidy [9, 10]. Patients with risk factors like age >18 months, high tumor stage, *MYCN* amplification (MNA), segmental chromosomal aberrations, and/or unfavourable histology fall into the high-risk group with poor prognosis [10]. High-risk NBLs often infiltrate adjacent organs and metastasize to regional lymph nodes, bone marrow (BM), bone, or soft tissue [10]. About 40–50% of NBLs present metastatic disease at diagnosis; however, in a subgroup of infants, spontaneous regression without any treatment might occur [11].

Numerical chromosomal changes are present in low- and intermediate-risk NBLs, whereas structural chromosomal alterations are strongly associated with more aggressive high-risk disease [12]. Chromosomal instability causes CNVs throughout the genome which, together with MNA, presents the most prominent clinically relevant features in the biologic and genomic landscape of NBL [13]. Segmental CNVs frequently comprise losses of chromosomes 1p, 3p, 4p, 11q and gains of 1q, 2p, 17q, but other loci may also be affected [14–16]. A rare but small group of familial NBL exists, being estimated to cover around 1–2% of all cases [17]. So far, mutations of only two genes have been identified as disease-causing in hereditary NBL: the paired-like homeobox 2B gene (*PHOX2B*), a key enzyme in early sympathic neurogenesis [18] and a tumor suppressor in NBL metastasis, and the anaplastic lymphoma kinase (*ALK*) gene, playing a role in both familial and sporadic NBLs [5, 19, 20]. *ALK* codes for a tyrosine kinase and appears as a promising therapeutic target [21, 22]. Apart from the activating mutations of *ALK* and the inactivating mutations in the α-thalassaemia/mental retardation syndrome X-linked (*ATRX*) gene are recurrent mutations infrequent in primary NBL [5]. Additionally, telomerase reverse transcriptase (*TERT*) gene rearrangements constitute a frequent genetic failure in NBLs being associated with poor outcome in high-risk patients [5, 15, 23, 24]. The *TP53* gene is involved in many cellular processes and is mutated in over 50% of all human cancers [25]. In NBL, the *TP53* mutation rate is only about 2%; however, protein accumulation is a frequent phenomenon both in NBL tumors and cell lines [26, 27]. One study reported p53 as a direct transcriptional target of *MYCN* in NBL [26]. Furthermore, mutations in genes involved in the *TP53* pathway may be biomarkers for a subgroup of NBLs with very high-risk within the larger group of high-risk tumors characterized by either classical (*MYCN/TERT*) or alternative (*ATRX*) telomere maintenance mechanisms [28, 29].

A number of research projects have explored the clinical potential of high-throughput sequencing technologies in adult cancers [30]. However, childhood cancers significantly diverge from adult cancers in terms of clinical behaviour, frequency, histopathology, genetic subtypes, and tumor biology [31, 32]. The extreme heterogeneous nature of NBL is challenging. Many studies have utilized sequencing technology to enhance the knowledge about NBL [5, 15, 33–35]. The presence of *ALK* mutations, as the most frequent among primary NBLs, has already been proven; however, other studies revealed novel alterations developed in relapsed NBLs, associated with activation of the *ALK-RAS-MAPK* pathway or mesenchymal transition [33, 36, 37].

In this study, whole exome sequencing (WES) was performed on paired tumor-normal samples from 18 Norwegian NBL patients: 16 high-risk, one intermediate (IR), and one very low risk (VLR), to investigate their mutational profile and to identify possible novel somatic variants. In addition, for six patients, WES on paired normal—relapse tumor samples was performed. Variants of genes known to be important for NBL development and aggressiveness were detected with a mean number of non-synonymous variants of 28 (range 3–346). Hotspot mutations in the *ALK* gene were identified in tumor samples from five of these high-risk NBL patients. Mutations in genes previously reported playing a role in NBL disease development were found, including *PHOX2B*, *TP53*, *DMD*, *ROS*, *LMO3*, *PRUNE2*, and *ERBB3*, members of the MAPK family and *ABCA2* genes. Our patient samples revealed few recurrent mutations; however, in addition to the *ALK* gene, variants in nine biologically relevant genes were identified being mutated in more than one patient i.e. *TMEM14B*, *TTN*, *FLG*, *RHBG*, *SHROOM3*, *UTRN*, *HLA-DRB1*, *OR6C68*, and *XIRP2*. Some potentially important genes were detected by pathway analysis and we hypothesize that further studies of their functional role in the origin and progression of NBL could lead to the discovery of new potential biomarkers.

## Results

A total of 16 primary and four relapse tumor samples of patients diagnosed with high-risk NBL were investigated by WES. Two additional samples of patients not fulfilling the clinical INRG high-risk disease criteria were also included in the study: patient 7 was diagnosed with a localized tumor without *MYCN* amplification but with an 11q deletion (intermediate risk) and experienced two relapses at different time points, and patient 15 was classified as stage MS with very low risk (no MNA, no 11q deletion), but displayed two segmental aberrations (+2p and +17q). For patient 23, a sample collected at time of diagnosis was not available. All tissue samples were taken prior to therapy, except the primary tumor of patient 14, which was collected after initial chemotherapy treatment had started. The median age at time of diagnosis was 44 months (range 1.5–192 months). Five patients were under the age of 18 months at the time of diagnosis and one of them was *MYCN* amplified. Among 18 patients at the age of ≥18 months, four were *MYCN* amplified and eight showed an 11q deletion (Table 1). Eight patients relapsed between 10 months and 3 years after diagnosis; seven patients died from the disease. They all belonged to the group of ≥18 months.

**Table 1:** Clinical characteristics of the NBL patients included in this study.

**Patient ID**	**1**	**2**	**4**	**5**	**6**	**7**	**8**	**9**	**11**	
**Gender**	M	M	F	M	M	M	M	M	M	
**Risk Stratification**	HR	HR	HR	HR	HR	IR	HR	HR	HR	
**LDH**	358	458	448	295	348	230	329	270	656	
**Genomic profile**	11q-	MNA	-	11q-	11q-	11q-	11q-	-	11q-	
**Age at diagnosis (months)**	97	1.5	192	69	84	72	18	23	43	
**Primary tumor site**	Abdomen	Adrenal gland	Abdomen	Cervical lymph node metastasis at diagnosis	Abdomen	Adrenal gland	Cervical lymph node metastasis at diagnosis	Abdomen	Adrenal gland	
**Metastatic site**	Skeleton, pancreas, lungs	Liver, BM	Skeleton, lymph nodes, BM	Skeleton, lymph nodes, BM	-	-	Skeleton, lymph nodes, BM	Skeleton, lymph nodes, BM	Skeleton, BM	
**Refractory disease**	No	No	No	Yes (BM)	Yes	No	No	Yes	No	
**Relapse**	Yes	No	No	No	NA	Yes	No	No	Yes	
**Time to relapse (months)**	36	-	-	-	-	13	-	-	10	
**Death**	Yes	No	No	No	Yes	Yes	No	No	Yes	
**Time to death (months)**	90	-	-	-	72	60	-	-	18	
**Patient ID**	**12**	**14**	**15**	**16**	**17**	**18**	**20**	**21**	**22**	**23**
**Gender**	F	F	M	F	F	M	M	M	F	F
**Risk Stratification**	HR	HR	VLR	HR	HR	HR	HR	HR	HR	HR
**LDH**	338	333	364	7969	858	1670	1674	3976	554	1884
**Genomic profile**	11q-	NA	-	MNA	11q-	MNA	MNA	-	-	-
**Age at diagnosis (months)**	18	17	4	127	44	141	146	47	14	4
**Primary tumor site**	Abdomen	Adrenal gland	Liver metastasis at diagnosis	Abdomen	Abdomen	Adrenal gland	Abdomen	Liver metastasis at diagnosis	Adrenal gland	Relapse in cranium (regio parietalis)
**Metastatic site**	Skeleton, BM	Skeleton lymph nodes, BM	Liver, BM	Skeleton, lymph nodes, BM	SSkeleton, lymph nodes, BM, liver, lungs	Liver, BM	Skeleton, lymph nodes, BM	Skeleton, lymph nodes, BM, liver, kidney	Skeleton, lymph nodes, BM	Liver, BM
**Refractory disease**	Yes	No	No	Yes	No	No	No	No	Yes	
**Relapse**	No	No	No	Yes	Yes	No	No	Yes	no	
**Time to relapse (months)**	-	-	-	-	18	-	-	16	-	-
**Death**	No	No	No	Yes	Yes	No	No	Yes	No	
**Time to death (months)**	-	-	-	7	96	-	-	24	-	-

F, female; M, male; HR, high-risk; VLR, very low risk; IR, intermediate risk; MNA, *MYCN*-amplification; and BM, bone marrow.

### Variants detected by WES

**Fig 1:**
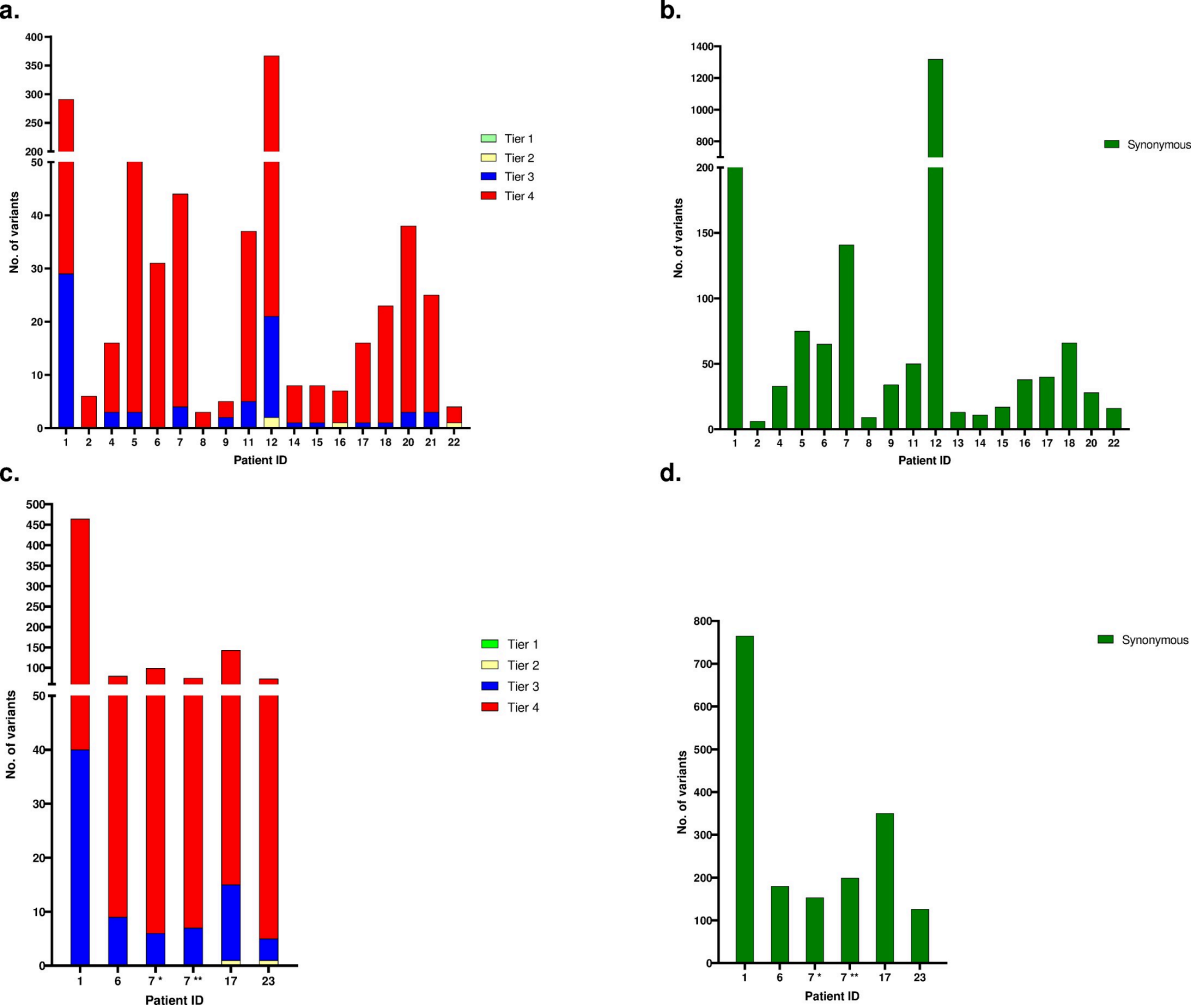
Total number of variants. Columns illustrate the total number of variants classified into Tiers 1, 2, 3, 4, and synonymous variations detected in primary (1a –b), and relapse samples (1c –d) of NBL patients included in the study. No Tier 1 variants were detected. For patient 7, two relapse samples (7 * and 7 **), collected at different time points and analyzed separately, are presented.

The total number of detected variants in the primary tumor samples revealed extreme variations between individual patients, ranging from 12 variants in patient 2 to 1687 variants detected in patient 12. The average number of variants for patients of ≥18 months is 237, while for those below 18 months was 14 variants. The mean and median numbers of total variants were 190 and 54, respectively. The mean and median numbers of all detected non-synonymous variants were 28 and 5, ranging from 1 to 346. The total numbers of variants detected in both, primary and relapse tumor samples, were classified into different Tiers (Fig 1A and 1B and S1 and S2 Tables). For the relapsed samples, the mean and median numbers of all detected non-synonymous variants were 66 and 27, ranging from 1 to 424. Numbers of variants detected in relapse samples were higher compared to the corresponding primary tumor samples (S3 Table). The average tumor cell percentage in all samples was 79 (60–90) (S6 Table). For all detected variants, the tumor allele frequency (TAF) is provided (S7 Table).

No Tier 1 variants with *strong clinical significance* for the user-specified cancer type have been identified in our study. Tier 2 variants were found in primary and relapse samples and included six variants in four genes, three in *ALK*, one in the *FLT3*, one in *KRAS*, and one in *PIK3CA* (Table 2). The *PIK3CA* gene was not mutated in the primary tumor of patient 17. For patient 23, no material from the primary tumor was available.

**Table 2:** Detected variants of genes classified according to potential clinically significance (Tier 2).

Patient ID	Gene name	Variant
**12**	*ALK*	p.Phe1174Leu
		c.3522C>A
**22**	*ALK*	p.Arg1275Gln
		c.3824G>A
23 *	*ALK*	p.Arg1275Gln
		c.3824G>A
**12**	*FLT3*	p.Thr22Met
		c.680C>T
**16**	*KRAS*	p.Gly12Val
		c.35G>T
17 *	*PIK3CA*	p.His1047Arg
		c.3140A>G

*, relapse sample.

Based on the Personal Cancer Genome Reporter (PCGR) report, we were able to identify *ALK* mutations in five patients. Three were classified as being clinically significant (Table 2), while the remaining ones had an uncertain clinical impact (Tier 3) for patient 9 (p.Phe1174Val, c.3520T>G) and patient 20 (p.Phe1174Leu, c.3522C>G). These four different missense variants of the *ALK* gene are all detected at positions (F1174 and R1275), known as hotspots in NBL.

Tier 3 includes 108 variants in 101 different genes classified as tumor suppressors and proto-oncogenes in the primary tumor samples of 12 patients (11 patients were ≥ 18 months) and 77 variants in 71 genes in six relapsed samples (Table 3A and 3B). Several of these variants have previously been reported to play a potential role in NBL disease development, including *TP53*, *DMD*, *ROS*, *LMO3*, *PRUNE2*, and *ERBB3*, members of the MAPK family (*MAP2K4* and *MAP2K7*) and *ABCA2* genes. Additionally, two variants in the *PHOX2B* gene were found (classified as Tier 4): one intron variant in patient 12 and one missense variant in patient 6.

**Table 3:** Variants classified into Tier 3.

**a**						
**Primary tumors**						
**Patient ID**	**Tumor suppressors**			**Proto-oncogenes**		
	**Gene**	**Variant**		**Gene**	**Variant**	
1	***GLIPR1***	p.Thr234Asn	c.701C>A	***AKR1B10***	p.Ala209Thr	c.625G>A
	***DLC1***	p.Gln1326Ter	c.3976C>T	***MYCL***	p.Arg214Gln	c.641G>A
	***BTG1***	p.Trp56Ter	c.167G>A	***MYBL1***	p.Asp263Gly	c.788A>G
	***PRUNE2***	p.Ala2067Thr	c.6199G>A	***NUAK1***	p.Ser499Asn	c.1496G>A
	***PRKDC***	p.Val646Met	c.1936G>A	***EHMT2***	p.Arg1053Cys	c.3157C>T
	***MUC2***	p.Gln736Ter	c.2206C>T	***CDH2***	p.Ala30Thr	c.88G>A
	***MUC2***	p.Ser1698Thr	c.5092T>A	***ITGB1***	p.Arg240His	c.719G>A
	***MAP2K4***	p.Glu221Ter	c.661G>T	***IQGAP1***	p.Thr292Met	c.875C>T
	***CREBBP***	p.Val2376Ile	c.7126G>A	***LMO3***	p.Gln86Ter	c.256C>T
	***ZFHX3***	p.Met887Thr	c.2660T>C	***USP15***	p.Arg429Ter	c.1285C>T
	***FBLN1***	p.His671Tyr	c.2011C>T	***MN1***	p.Asp1020Gly	c.3059A>G
	***TCHP***	p.Arg285His	c.854G>A	***PDGFB***	p.Thr169Met	c.506C>T
	***FBP1***	p.Ala5Val	c.14C>T	***WNT10A***	p.Ala148Val	c.443C>T
	***DMD***	p.Ser42Gly	c.124A>G	***IRS2***	p.Ser731Ala	c.2191T>G
	***-***	-	-	***G6PD***	p.Arg469His	c.1406G>A
4	***STAG2***	p.Glu1147Val	c.3440A>T	***SETBP1***	p.Asp341Val	c.1022A>T
	***-***	-	-	***EIF3A***	p.Arg611Met	c.1832G>T
5	***DKK1***	p.Asp142Tyr	c.424G>T	***FASN***	p.Gly950Ser	c.2848G>A
	***-***	-	-	***OTX2***	p.Arg55Leu	c.164G>T
7	***FAS***	-	c.569-1G>C	***AFP***	p.Ala439Ser	c.1315G>T
	***RP1***	p.Met508Leu	c.1522A>T	***HOXA1***	p.Gly122Trp	c.364G>T
9	***PHLPP1***	p.Gly1619Glu	c.4856G>A	***ALK***	p.Phe1174Val	c.3520T>G
11	***TP53***	p.Gly199Val	c.596G>T	***HMMR***	p.Leu109Met	c.325C>A
	***BCL6B***	p.Gly35Glu	c.104G>A	***PGR***	p.Ser796Ter	c.2387C>A
	***MAP2K7***	p.Pro286Ala	c.856C>G	*-*	-	-
12	***PRUNE2***	p.Thr1004Met	c.3011C>T	***ROS1***	p.Ser1109Leu	c.3326C>T
	***KANK1***	p.Glu432Gln	c.1294G>C	***ERBB3***	p.Ser1119Cys	c.3355A>T
	***E2F2***	p.Gln226His	c.678G>T	***MYBL2***	p.Ile624Met	c.1872C>G
	***PHLPP1***	p.Glu931Lys	c.2791G>A	***KIF14***	p.Met753Leu	c.2257A>T
	***CDH11***	p.Met275Ile	c.825G>A	***USP6***	p.Arg912Gln	c.2735G>A
	***CBX4***	p.Pro535Ala	c.1603C>G	***TNK2***	p.Arg1086His	c.3257G>A
	***SUSD2***	p.Asn466Ser	c.1397A>G	***ATF7IP***	p.Asn348Ile	c.1043A>T
	***RIT1***	p.Glu11Gln	c.31G>C	***MMP9***	p.Arg668Gln	c.2003G>A
	***MCC***	p.Ser25Gly	c.73A>G	***UBD***	p.Ile68Thr	c.203T>C
	***SUN2***	p.Leu89Arg	c.266T>G	***DBH***	p.Arg549Cys	c.1645C>T
	***DMD***	p.Asp882Gly	c.2645A>G	*-*	-	-
13	*-*	-	-	***USP39***	p.Leu446Phe	c.1338G>T
14	***PLXNC1***	p.Gly964Glu	c.2891G>A	*-*	-	-
16	*-*	-	-	***NFKB1***	-	c.1300+1G>A
17	***DMBT1***	p.Val1545Leu	c.4633G>C	***PHF20***	p.Ser880Phe	c.2639C>T
	*-*	-	-	***ABCA2***	p.Asp578His	c.1732G>C
	*-*	-	-	***WDR7***	p.Thr1076Arg	c.3227C>G
	*-*	-	-	***FCRL1***	p.Thr160Ile	c.479C>T
	*-*	-	-	***AP3S1***	p.Pro158Leu	c.473C>T
	*-*	-	-	***ISOC2***	p.Arg49Trp	c.145C>T
	*-*	-	-	***OPLAH***	p.Asn1105Ile	c.3314A>T
	*-*	-	-	***ODF2***	p.Leu751Met	c.2251C>A
	*-*	-	-	***UBQLN2***	p.Asp314Asn	c.940G>A
	*-*	-	-	***COL5A1***	p.Ala397Val	c.1190C>T
	*-*	-	-	***MTNR1B***	p.Arg154His	c.461G>A
	*-*	-	-	***PSD3***	p.Ala1003Ser	c.3007G>T
	*-*	-	-	***ALMS1***	p.Gln1155Glu	c.3463C>G
	*-*	-	-	***DSE***	p.Val592Asp	c.1775T>A
	*-*	-	-	***UNC13A***	p.Ser1562Phe	c.4685C>T
	*-*	-	-	***MAP7D1***	p.Gln63Pro	c.188A>C
	*-*	-	-	***RETSAT***	p.Gly536Arg	c.1606G>A
	*-*	-	-	***WDR75***	p.Val486Phe	c.1456G>T
	*-*	-	-	***TMEM14B***	p.Arg108Cys	c.322C>T
	*-*	-	-	***WDR91***	p.His572Asn	c.1714C>A
	*-*	-	-	***ZNRF4***	p.Glu315Ter	c.943G>T
	*-*	-	-	***IGLJ3***	p.Val35Leu	c.103G>T
18	***MEF2D***	p.Pro460Gln	c.1379C>A	***SULF2***	p.Trp735Leu	c.2204G>T
	***RP1***	p.Leu2115Phe	c.6345A>C	*-*	-	-
20	***TP53BP1***	p.Gly1788Glu	c.5363G>A	***ALK***	p.Phe1174Leu	c.3522C>G
	*-*	-	-	***RSF1***	p.Glu311Ter	c.931G>T
**b**						
**Relapses**						
**Patient ID**	**Tumor suppressors**			**Protooncogenes**		
	**Gene**	**Variant**		**Gene**	**Variant**	
1	***CDC73***	p.Val442Ile	c.1324G>A	***MAPK7***	p.Glu783Lys	c.2347G>A
	***INPP4B***	p.Lys782Asn	c.2346G>T	***MYCL***	p.Arg214Gln	c.641G>A
	***TMPRSS11A***	p.Gln240Arg	c.719A>G	***RET***	p.Arg694Gln	c.2081G>A
	***KANK1***	p.Asp29Asn	c.85G>A	***NUP214***	p.Gly1727Arg	c.5179G>A
	***PRKDC***	p.Val646Met	c.1936G>A	***IRF4***		c.-56+1G>A
	***PTPN13***	p.Leu1225Met	c.3673C>A	***PHGDH***	p.His532Arg	c.1595A>G
	***PML***	p.Arg670Cys	c.2008C>T	***CTTN***	p.Gly440Asp	c.1319G>A
	***DNMT3A***	p.Pro569Leu	c.1706C>T	***CDH2***	p.Ala30Thr	c.88G>A
	***AKT1***	p.Arg328Cys	c.982C>T	***WBP2***	p.Gly151Arg	c.451G>A
	***PRSS21***	p.Ala224Val	c.671C>T	***GTPBP4***	p.Thr189Met	c.566C>T
	***PLK2***	p.Asp443Asn	c.1327G>A	***EIF3A***	p.Arg959Gln	c.2876G>A
	***PLK2***	p.Arg174Ile	c.521G>T	***ITGB1***	p.Arg240His	c.719G>A
	***SULF1***	p.Met255Thr	c.764T>C	***LMO3***	p.Gln86Ter	c.256C>T
	***ZFHX3***	p.Arg2712Ser	c.8136A>T	***USP15***	p.Arg429Ter	c.1285C>T
	***FBLN1***	p.His671Tyr	c.2011C>T	***KDM5C***	p.Arg909Trp	c.2725C>T
	***LMO7***	p.Arg119Cys	c.355C>T	***FOXC1***	p.Gln530Arg	c.1589A>G
	***PCDH20***	p.Ala769Thr	c.2305G>A	***WNT10A***	p.Ala148Val	c.443C>T
	***NOTCH3***	p.Gly2052Asp	c.6155G>A	***GLI3***	p.Leu68Phe	c.202C>T
	***ARHGAP10***	p.Ile332Val	c.994A>G	***MEF2C***	p.Ser350Ile	c.1049G>T
	***FBP1***	p.Ala5Val	c.14C>T	*-*		
	***PHLDA2***	p.Gly29Val	c.86G>T	*-*		
6	***DLEC1***	p.Pro1028Arg	c.3083C>G	***FOXQ1***	p.Arg287Gly	c.859A>G
	***ZEB2***	p.Pro922Arg	c.2765C>G	***RELB***	p.Val230Glu	c.689T>A
	***SMARCA2***	p.Leu1320Phe	c.3958C>T	***IRS1***	p.Lys61Ter	c.181A>T
	***BACH2***	p.Gln722His	c.2166G>T	***-***	-	**-**
	***MEF2D***	-	c.1248-1G>A	***-***	-	**-**
	***ALOX15***	p.Phe203Ser	c.608T>C	***-***	-	**-**
7 *	***VWa5A***	p.Pro638Leu	c.1913C>T	***MED12***	p.Gly44Ser	c.130G>A
	***DUSP10***	p.Lys185Arg	c.554A>G	***MET***	p.Pro475Ser	c.1423C>T
	***NR1H4***	p.Ser164Arg	c.492C>A	***-***	-	**-**
	***TES***	p.Ala99Ser	c.295G>T	***-***	-	**-**
7 **	***TUSC2***	p.Gln74Lys	c.220C>A	***MED12***	p.Gly44Ser	c.130G>A
	***DUSP10***	p.Lys185Arg	c.554A>G	***TGFB1***	-	c.356-2A>G
	***LIF***	p.Ter203GlnextTer33	c.607T>C	***ADAR***	p.Leu1067Phe	c.3201G>T
	***NR1H4***	p.Ser164Arg	c.492C>A	***-***	-	**-**
17	***ING1***	p.Ser362Pro	c.1084T>C	***FOXM1***	p.Ser472Pro	c.1414T>C
	***TUSC3***	p.Leu29Pro	c.86T>C	***PELP1***	p.Gly179Val	c.536G>T
	***USP9X***	-	c.6210-1G>T	***SMYD3***	p.Lys94Glu	c.280A>G
	***PTPRJ***	p.Tyr182His	c.544T>C	***RET***	p.Leu132Pro	c.395T>C
	***PTPRG***	p.Met862Val	c.2584A>G	***MED12***	p.Ala860Thr	c.2578G>A
	***NOTCH2***	p.Ile707Thr	c.2120T>C	***HK2***	p.Val412Ala	c.1235T>C
	***-***	-	**-**	***RAB22A***	p.Phe93Leu	c.277T>C
	***-***	-	**-**	***AHI1***	p.Ser1108Gly	c.3322A>G
23	***ESRP1***	p.Tyr557Ter	c.1671C>A	***GLI2***	p.Al1364Thr	c.4090G>A
	***-***	**-**	-	***SLC2A1***	p.Pro187Thr	c.559C>A
	***-***	**-**	-	***EIF3***	p.Gly40Val	c.119G>T

Genes with detected variants in patients 1, 4, 5, 7, 9, and 11, 12, 14, 16,17, 18, and 20 at the time of diagnosis, classified into Tier 3.

Genes with detected variants in patients 1, 6, 7, 9, 17, and 23 at the time of relapse, classified into Tier 3. Samples 7 * and 7 ** are two subsequent relapses in the same patient; -, not applicable

To evaluate the cancer-associations of certain detected variants, the MutationAssessor predictor algorithm was applied. This sequence-based tool uses the impact of mutations to rank genes according to their significance for cancer. Taking into account the functional impact of amino-acid substitutions on proteins, it gives a functional impact score (FIS) for every non-synonymous mutation. If the FIS is >2.00, the mutation is considered to have a damaging effect [38, 39]. The *PHLPP1* gene was mutated in two of the patients with the prediction of a damaged protein variant c.4856G>A in patient 9 and tolerated c.2791G>A variant in patient 12. Beside the observed *PHLPP1* mutations, additional genes related to the RET signaling pathway were detected and classified into Tier 3, both in primary and relapse tumor samples: *ERBB3*, *MET*, *PDGFB*, *RET*, *IRS2*, *DUSP10*, *AKT1*, *RIT1*, *MAPK7*, *NFKB1*, *MAP2K7*, *MAP2K4*, *MEF2C*, and *MMP9*.

Variants classified into Tier 3 were grouped into mutations detected only in the primary tumor, shared by the primary and relapse tumor, or unique for relapse samples. Only patient 1 revealed shared variants in nine genes, with TAFs ranging from 14 to 83% (S7 Table); however, additional 20 and 31 gene variants were found to be unique for the primary and relapse, respectively (Table 4). The other three patients with both the primary and relapse tumor samples did not share gene variants classified into Tier 3. We observed shared mutated variants of *DUSP10*, *NR1H4*, and *MED12* genes between two relapses of patient 7 (Table 5). *RET* gene variants were detected in two of the relapse samples, patient 1 and 17; however, at different positions (c.2081G>A; c.395T>C).

**Table 4:** Variants detected in patient 1.

Patient ID	PT			PT & RT			RT		
	Gene	Variant		Gene	Variant		Gene	Variant	
1	***GLIPR1***	p.Thr234Asn	c.701C>A	***PRKDC***	p.Val646Met	c.1936G>A	***CDC73***	p.Val442Ile	c.1324G>A
	***DLC1***	p.Gln1326Ter	c.3976C>T	***FBLN1***	p.His671Tyr	c.2011C>T	***INPP4B***	p.Lys782Asn	c.2346G>T
	***BTG1***	p.Trp56Ter	c.167G>A	***FBP1***	p.Ala5Val	c.14C>T	***TMPRSS11A***	p.Gln240Arg	c.719A>G
	***PRUNE2***	p.Ala2067Thr	c.6199G>A	***MYCL***	p.Arg214Gln	c.641G>A	***KANK1***	p.Asp29Asn	c.85G>A
	***MUC2***	p.Gln736Ter	c.2206C>T	***CDH2***	p.Ala30Thr	c.88G>A	***PTPN13***	p.Leu1225Met	c.3673C>A
	***MUC2***	p.Ser1698Thr	c.5092T>A	***ITGB1***	p.Arg240His	c.719G>A	***PML***	p.Arg670Cys	c.2008C>T
	***MAP2K4***	p.Glu221Ter	c.661G>T	***LMO3***	p.Gln86Ter	c.256C>T	***DNMT3A***	p.Pro569Leu	c.1706C>T
	***CREBBP***	p.Val2376Ile	c.7126G>A	***USP15***	p.Arg429Ter	c.1285C>T	***AKT1***	p.Arg328Cys	c.982C>T
	***ZFHX3***	p.Met887Thr	c.2660T>C	***WNT10A***	p.Ala148Val	c.443C>T	***PRSS21***	p.Ala224Val	c.671C>T
	***TCHP***	p.Arg285His	c.854G>A	-	-	-	***PLK2***	p.Asp443Asn	c.1327G>A
	***DMD***	p.Ser42Gly	c.124A>G	-	-	-	***PLK2***	p.Arg174Ile	c.521G>T
	***AKR1B10***	p.Ala209Thr	c.625G>A	-	-	-	***SULF1***	p.Met255Thr	c.764T>C
	***MYBL1***	p.Asp263Gly	c.788A>G	-	-	-	***LMO7***	p.Arg119Cys	c.355C>T
	***NUAK1***	p.Ser499Asn	c.1496G>A	-	-	-	***PCDH20***	p.Ala769Thr	c.2305G>A
	***EHMT2***	p.Arg1053Cys	c.3157C>T	-	-	-	***NOTCH3***	p.Gly2052Asp	c.6155G>A
	***IQGAP1***	p.Thr292Met	c.875C>T	-	-	-	***ARHGAP10***	p.Ile332Val	c.994A>G
	***MN1***	p.Asp1020Gly	c.3059A>G	-	-	-	***PHLDA2***	p.Gly29Val	c.86G>T
	***PDGFB***	p.Thr169Met	c.506C>T	-	-	-	***MAPK7***	p.Glu783Lys	c.2347G>A
	***IRS2***	p.Ser731Ala	c.2191T>G	-	-	-	***RET***	p.Arg694Gln	c.2081G>A
	***G6PD***	p.Arg469His	c.1406G>A	-	-	-	***NUP214***	p.Gly1727Arg	c.5179G>A
	-	-	-	-	-	-	***IRF4***		c.56+1G>A
	-	-	-	-	-	-	***PHGDH***	p.His532Arg	c.1595A>G
	-	-	-	-	-	-	***CTTN***	p.Gly440Asp	c.1319G>A
	-	-	-	-	-	-	***WBP2***	p.Gly151Arg	c.451G>A
	-	-	-	-	-	-	***GTPBP4***	p.Thr189Met	c.566C>T
	-	-	-	-	-	-	***EIF3A***	p.Arg959Gln	c.2876G>A
	-	-	-	-	-	-	***KDM5C***	p.Arg909Trp	c.2725C>T
	-	-	-	-	-	-	***FOXC1***	p.Gln530Arg	c.1589A>G
	-	-	-	-	-	-	***GLI3***	p.Leu68Phe	c.202C>T
	-	-	-	-	-	-	***MEF2C***	p.Ser350Ile	c.1049G>T

PT, variants unique for primary tumor sample; PT and RT, variants shared between PT and RT samples of patient 1; RT, variants unique for the relapse sample, and -, not applicable

**Table 5:** Variants detected in patient 7.

**Patient ID**	**PT**			**PT & RT1**			**RT1**		
	**Gene**	**Variant**		**Gene**	**Variant**		**Gene**	**Variant**	
7	***FAS***	-	c.569-1G>C	-	-	-	***VWa5A***	p.Pro638Leu	c.1913C>T
	***RP1***	p.Met508Leu	c.1522A>T	-	-	-	***DUSP10***	p.Lys185Arg	c.554A>G
	***AFP***	p.Ala439Ser	c.1315G>T	-	-	-	***NR1H4***	p.Ser164Arg	c.492C>A
	***HOXA1***	p.Gly122Trp	c.364G>T	-	-	-	***TES***	p.Ala99Ser	c.295G>T
		-	-	-	-	-	***MED12***	p.Gly44Ser	c.130G>A
		-	-	-	-	-	***MET***	p.Pro475Ser	c.1423C>T
7	**RT1**			**RT1 & RT2**			**RT2**		
	**Gene**	**Variant**		**Gene**	**Variant**		**Gene**	**Variant**	
	***VWa5A***	p.Pro638Leu	c.1913C>T	***DUSP10***	p.Lys185Arg	c.554A>G	***TUSC2***	p.Gln74Lys	c.220C>A
	***TES***	p.Ala99Ser	c.295G>T	***NR1H4***	p.Ser164Arg	c.492C>A	***LIF***	p.Ter203GlnextTer33	c.607T>C
	***MET***	p.Pro475Ser	c.1423C>T	***MED12***	p.Gly44Ser	c.130G>A	***TGFB1***	-	c.356-2A>G
		-	-		-	-	***ADAR***	p.Leu1067Phe	c.3201G>T

PT, variants unique for primary tumor sample; PT and R1, variants shared between PT and the first relapse of patient 7; RT1, variants unique for the first relapse sample; RT2, variants unique for the second relapse; RT1 & RT2, variants shared by RT and RT2; and -, not applicable.

Patient samples 2, 6, 8, and 15 exhibited a general lower mutation burden of 12, 96, 12, and 24 detected variants; and neither Tier 2 nor 3 variants were detected. Oncoscore was applied to investigate further potential targets in Tier 4 classified variants in these patients. In patient 2, a total of 12 mutations were found, whereof six were classified as Tier 4 with the highest reported Oncoscore value at ~0.26 in a non-synonymous variant of the *NCKAP5* gene, predicted to cause alterations of the resulting protein. All other identified non-synonymous mutations exhibited an Oncoscore below ~0.27. In patient 6, 31 out of a total of 96 mutations were classified as Tier 4. A non-synonymous variant was detected for the *TNIK* gene, predicted to produce a damaged protein, revealing an Oncoscore of ~0.624. Additionally, mutated variants of *PHOX2B* gene were detected with Oncoscore ~0.26 (p.Glu129Ter c.385G>T). Unfortunately, MutationAssessor does not provide prediction assessment for this variant. In the tumor sample of patient 8, a total of 12 mutations were determined, of which three were classified into Tier 4. A non-synonymous variant of the *PSMC3* gene was found and reported as predicted to produce a malformed protein but with a low Oncoscore of ~0.27. In patient 15, 24 mutations were detected, seven of them being classified as Tier 4. One variant found in the *AHNAK2* gene was previously reported as being destructive resulting in a changed protein with an Oncoscore of ~0.60 (S5 Table).

### Identification of potentially damaging genes and variants

WES analysis of all samples included in this study revealed a total of 3426 variants. To select a dataset of more biologically relevant variations, filtering was performed using the MutationAssessor results from PCGR. This filtering step reduced the total number of events to 320 potential pathogenic variants. Among these variants, only so far unreported genes mutated in more than one patient are presented (thus, known hotspot genes like *ALK* were excluded).

Based on the filtering described above, 17 biologically relevant variants in nine biologically relevant genes were identified in nine patients: *TMEM14B*, *HLA-DRB1*, *OR6C68*, *TTN*, *FLG*, *RHBG*, *SHROOM3*, *UTRN*, and *XIRP2*. Two genes with identical variants were present in two patients (*TMEM14B* and *HLA-DRB1*) with a TAF ranging from 44 to 58%, and seven genes with different variants were identified (Tables 6 and S7). Two patients (7 and 12) with mutations of the *OR6C68* gene at 12q13.2 showed copy number gains of the corresponding part of 12q (*unpublished data*). We observed a tendency to obtain mutated variants more frequently in the primary tumor samples of patient 1 and 12.

**Table 6:** Genes with non-synonymous variants detected in primary tumor samples, predicted to be damaging by the MutationAssessor program, found in more than one patient.

Gene name	Chromosome location	SCA region	Protein	Patient ID	CNV
***RHBG***	1q22	c.251G>A	p.Arg84His	1	0
		c.428T>A	p.Val143Asp	12	0
***SHROOM3***	4q21.1	c.905C>T	p.Ala302Val	1	0
		c.3160G>T	p.Val1054Leu	12	0
		c.3869C>T	p.Pro1290Leu		
***TTN***	2q31.2	c.101809C>T	p.His33937Tyr	1	0
		c.78674T>C	p.Ile26225Thr	12	0
***FLG***	1q21.3	c.1815G>T	p.Gln605His	1	0
		c.3176G>T	p.Arg1059Ile	5	0
***UTRN***	6q24.2	c.1614G>T	p.Gln538His	1	0
		c.1967T>G	p.Val656Gly	4	0
***HLA-DRB1***	6p21.32	c.654A>T	p.Arg218Ser	5	0
				12	0
***OR6C68***	12q13.2	c.416G>A	p.Cys139Tyr	7	gain/ampl
		c.456G>T	p.Met152Ile	12	gain/ampl
***XIRP2***	2q24.3	c.6168G>T	p.Leu2056Phe	11	0
		c.5402G>A	p.Arg180His	12	0
***TMEM14B***	6p24.2	c.322C>T	p.Arg108Cys	14	na
				17	0

SCA, structural chromosome abnormality; CNV, copy number variant; ampl, amplification, and na, not available.

### Gene ontology analysis

Gene ontology analysis (GOseq) for non-synonymous variants revealed several pathways with potential biological impact on NBL, including neuron cell-cell adhesion, biological adhesion, and PI3K/Akt signaling pathway, immune response-regulating cell surface receptors, or innate immune response activating cell surface receptors (Table 7). In these pathways, the *RET*, *PIK3CA*, *PHLPP1*, *KRAS*, *NFKB1*, *MUC5B*, and *MUC6* genes were found to be mutated in at least two patients (Table 5). Further signaling pathways with potential biological significance in NBL and details of the analysis are presented in S5 Table.

**Table 7:** Pathways with a potential biological impact on NBL verified by GOSeq analysis.

Pathways with a potential biological impact on NBL	Nr of patients with significant GO term	Genes
neuron cell-cell adhesion	4	*RET*
biological adhesion	4	*PIK3CA*, *RET*
PI3K/Akt signaling	4	*PHLPP1*
immune response-regulating cell surface receptor signaling	3	*KRAS*, *NFKB1*, *MUC5B*, *MUC6*, *PIK3CA*
innate immune response activating cell surface receptor signaling	2	*KRAS*, *NFKB1*, *MUC5B*, *MUC6*

### Genes reported with different ranking rules

In all of the investigated samples, a total of 2722 out of 20805 genes of the human reference genome 37 (grch37) revealed at least one mutation. To identify cardinal genes of NBL and to evaluate the identified mutated genes, different ranking rules were applied. First, genes were ranked according to the average number of mutations, resulting in a tendency to rank long genes higher. Next, ranking by average divided by gene length was performed and finally, genes were ranked by the total number of observations with mutations in the gene (Table 8). The *TTN* gene was reported among the top 10 under two different applied ranking rules. Besides *TTN*, there were no predominant findings detected except two genes listed (*TRIM9*, *PKHD1*) in top 10, previously reported in the context of NBL.

**Table 8:** Results of ranked genes according to different ranking rules.

G	nG	B
*OBSCN*	*THSD7B*	*TTN*
*LRRC8B*	*GBP4*	*LPO*
*SSPO*	*MIR519C*	*HPRT1*
*ASTAB1*	*RTN3*	*TNS1*
*TTN*	*ZFHX2*	*EPB41L2*
*DHRSX*	*CRB1*	*VAC14*
*ACE*	*NHSL1*	*MACF1*
*RASGEF1B*	*TRIM9*	*RHPN2*
*MIR4472-1*	*CAV2*	*KIF2B*
*ZBTB17*	*FOXS1*	*OBSCN*
*YWHAZ*	*-*	*PKHD1*
*PKD1L2*	*-*	*CSPG4*
*DNAH3*	*-*	*MUC16*
*PMPCA*	*-*	*AHNAK2*
*POU5F2*	*-*	*SSPO*
*MED25*	*-*	*DMD*
*DKK1*	*-*	*UPB1*
*DOCK5*	*-*	*LRRN4*
*RNF217-AS1*	*-*	*RABL2A*
*EEF1AKNMT*	*-*	*ADAM33*

G, ranking by average, nG; ranking by average normalized with exome length; B, ranking based on the total number of observations with mutations in the gene. *TTN* ranked under two different rules among the top 10. No other predominant findings were detected.

## Discussion

Different research groups have used sequencing and CNVs analysis in various efforts to explore the nature of NBL and to find novel strategies for prognostic assessment and therapeutic stratification of NBL with variable results [15, 28, 33, 40–43]. However, the remarkable heterogeneity of the disease makes it difficult to discover novel candidate genes, relevant for biological behaviour or targeted treatment. Moreover, this heterogeneity may increase under the treatment, and influence clinical interpretation of genetic findings and treatment strategy as pointed out recently [44]. In our study, we identified novel coding variants of genes possibly contributing to the understanding of these processes. Beside the confirmation of known mutations in *ALK*, we identified changes in genes of the RET signalling pathway, the RAS-MAPK and p53 signalling pathway, immune response genes, and other previously described NBL-related genes. We also detected specific features of the relapse tumor samples, several over-represented genes, and novel non-synonymous variants of genes occurring in more than one patient sample. Presumably, an increased allelic frequency correlates with the oncogenic potential of identified gene variants [45]. In our study, the average TAF for all detected variants is higher than 48%, supporting the assumption that the detected mutations could influence the NBL progression.

### *ALK* mutations and the RET signalling pathway

*ALK* activating mutations were identified in five patients, all at hotspot positions, that might be candidates for using *ALK*-targeted inhibitors [22]. Three of the patients carrying these mutations were above the age of 18 months at the time of diagnosis and one was exactly at the critical age of 18 months. In patient 9, an *ALK* mutation was found in addition to a mutated variant of the *PHLPP1* gene. *PHLPP1* is related to the RET signalling pathway and known for the promotion of tumor progression [46]. The *RET* gene is involved in neural crest development and ontogenesis of the enteric nervous system. Besides, *RET* is commonly expressed in NBL [47]. The *PHLPP1*- and additional genes of the RET signalling pathway were detected in six other primary and relapse tumor samples included in the study (Table 3). All of these patients were 18 months of age or older at time of diagnosis. Additionally, five out of seven patients presented an 11q deletion, one was MNA and for one there was no clinically relevant genomic changes detected. Our analysis detected two different variants of the *RET* gene in the relapsed samples of patient 1 and patient 17; however, they were classified by MutationAssessor as predicted to be tolerated. Moreover, in six patients GOSeq analysis identified neuron cell-cell adhesion and biological adhesion pathways, both pathways include the *RET* gene (Table 7).

### The RAS-MAPK and p53 signalling pathway

Mutations in the RAS-MAPK signalling pathway in patients with NBL are associated with poor prognosis [37, 48]. An example for this statement is patient 16, where all clinical characteristics typically indicate an unfavourable outcome of the disease: MNA, age >18 months at diagnosis, metastasis to skeleton, lymph nodes, and BM. This patient relapsed and died 7 months after diagnosis. So far, *KRAS* mutations have been linked mostly to relapses [37, 49], while WES results for patient 16 detected a *KRAS* variant in the primary tumor sample classified into Tier 2. Parallel to *ALK* as being an activator of RAS-MAPK pathway, there are other genes promoting its oncogenic function in NBL, like *PHOX2B* or *DMD* [48]. Both of these genes show mutated variants in a relapse sample of patient 6 and primary tumor samples of patient 1 and 12; all three patients present an 11q deletion and were above 18 months at the time of diagnosis.

Poor prognosis in NBL patients is also associated with mutations in genes of the p53 signalling pathway, e.g. *CREBBP* [28]. A mutated variant in the *CREBBP* gene (c.7126G>A) was detected in the primary tuomr of patient 1, presenting an 11q deletion and relapse disease.

### Previously described NBL-related genes

Additional mutations were detected in genes, previously reported to play a potential role in NBL disease development, including *TP53*, *DMD*, *ROS*, *LMO3*, *PRUNE2*, and *ERBB3*, members of the MAPK family (*MAP2K4* and *MAP2K7*) and the *ABCA2* gene [48, 50, 51]. In three patients with *MYCN* amplification mutated variants of *NFKB1*, *ALK*, and *SULF2* were detected. There is evidence that SULF2 is over-expressed in MNA NBL cell lines [52]. All three patients were older than 18 months at the time of diagnosis.

### Immune response genes

Immunotherapy with the anti-GD2 antibody is an important step of standard treatment protocol for high-risk NBL patients. It is based on inducing immune responses, e.g. by infusing monoclonal antibodies against the tumor-associated disialoganglioside *GD2*, combined with for example granulocyte–macrophage colony-stimulating factor and interleukin-2 [53]. Yet, 40% of NBL patients relapse [54]. Following GOSeq results, we identified mutations in genes involved in immune response-regulating cell surface receptor signalling pathways, e.g. mucines (*MUC5B*, *MUC6*, and *MUC16*), *KRAS*, *PIK3CA*, and *NFKB1* genes. All changes were found in the relapse samples, except the *KRAS* variant. Mutations in immune–response related genes detected in relapse tumor, followed by specific functional studies, could provide an important answer what differs in responders and non-responders of immunotherapy and why it is still not that effective. There is no reported association of genes of the *MUC* gene family with NBL, but these genes are known to play a role in cancer cells differentiation and proliferation, interacting, and regulating tumor microenvironment [55, 56].

### Specific features of the relapse tumor samples

The relapsed samples examined in our study exhibited more mutated variants in comparison to the primary tumor (both non-synonymous and synonymous), confirming previous findings [33]. This leads to genetic instability and diversity being a major obstacle in the research on prognostic markers and successful treatment in NBL. Five out of six patients who experienced a relapse presented an 11q deletion and one was MNA. All these patients died from the disease. Genes found mutated in relapse of patient 1, such as *LMO3* or *RET*, are associated with unfavourable outcome of the disease and tumor progression [57, 58]. The TAFs of these genes were 83 and 56%, respectively (S7 Table). Based on our findings, the clinical outcome of the patient and the published literature, we speculate that the detected mutations may have rendered these genes to become oncogenic drivers contributing to an unfavourable prognosis and disease development.

Recent studies suggest that *AKT* is a critical prognostic factor for NBL and that its expression is correlated with poor prognosis of the disease [59]. Additionally, we observed recurrent mutations in *DUSP10* in relapse samples of patient 7. *DUSP10* is a member of regulators of neuronal cell growth and differentiation [60]. Taking into account that the TAF of *DUSP10* amounted to 48%, and the tumor cell infiltration was 80%, indicates the possibilities of a driver oncogene in this relapse.

### Specific over-represented mutated genes

The purposes of this study were to discover novel mutations crucial for the origin, progression, or treatment on high-risk NBL. To this end, we asked whether certain mutations were over-represented in our samples. Despite the relatively low number of patients, we were able to find novel candidates. Due to the high diversity in the mutated genes, different ranking rules were applied in various analytic attempts. Of the top 10 candidates in the various lists, only the *TRIM9* and *PKHD1* genes have been previously reported in the context of NBL [61, 62].

### Novel non-synonymous variants of genes occurring in more than one patient

The biological relevance of non-synonymous variants of genes occurring in more than one patient and predicted to cause protein changes were analysed by the mutation effect predictor MutationAssessor algorithm. Here, we describe some interesting candidates detected in this group: ***TMEM14B*, *OR6C68*, *TTN*, *SHROOM3*,** *and* ***UTRN*.**

There is no record of *TMEM14B* being linked to NBL and the association of *TMEM14B* to cancer is not clear [33]; however, other members of the TMEM protein family have been found in some NBL samples. There is no clinical pattern between patients with mutated variant of *TMEM14B*, but one of two presents 11q deletion together with an age above 18 months at the time of diagnosis.

A possible association of ***OR6C68*** to cancer has so far not been described, but the expression of other members of the odorant receptor family genes is documented in olfactorial NBL [63], a central nerve-derived neoplasm, which does not belong to the family of sympathic peripheral neuroblastic tumors (PNTs). In our study, both patients with a detected variant of *OR6C68* have in addition important risk factors as an 11q deletion and age above or equal to 18 months at the time of diagnosis.

Titin (***TTN****)* is one of the longest genes in the human genome and therefore exposed to a higher risk of random mutations. Nevertheless, this pure statistical statement does not exclude the possibility of biologically relevant mutations in this gene [64]. Mutations in *TTN* have been previously observed in NBL and olfactorial NBL; however, no obvious conclusion of its biological function was described [65–67].

We observe mutated variants of ***SHROOM3*** in patients presenting an 11q deletion with additional detected mutations in *DMD*, *PRUNE2*, and *RHGB* genes. There is no established relation between *SHROOM3* and NBL, but *SHROOM3* has been identified as a strong candidate involved in the pathogenesis of craniofacial microsomia, which is a disease believed to be partially caused by disturbances of neural crest cells during embryogenesis [68]. NBL arise like other PNTs of sympathic origin from neural crest-derived cells. Thus, sequence variations of *SHROOM3* might occur, and a possible impact of NBL development is not unlikely. Mutations in *SHROOM3* have also been observed in relapsed acute lymphoblastic leukaemia [69].

Different non-synonymous variants for ***UTRN*** located on chromosome 6q were detected in two of the patients (1 and 4). We observed different variants of *EIF3A* gene in the primary tumor of patient 4 and the relapse sample of patient 1. The 6q24 region is commonly deleted in several types of cancers. Mutations in this region have previously been observed in NBL [70]. However, none of our patients with detected variants showed CNVs in that region (*unpublished data*).

Our findings demonstrate a remarkable divergence in both clinical and molecular characteristics of NBL, highlighting again the registered enormous heterogeneity observed in this disease.

In summary, we were able to confirm an unfavourable effect of mutations in RAS-MAPK, RET, or p53 signalling pathway genes. Moreover, some reported variants correlated with the occurrence of relapses and fatal outcome of the disease. In addition, we detected mutated variants of immune–response genes in the majority of relapse tumor samples. Primary refractory or relapsing disease significantly limits the survival of high-risk NBL patients.

Comparing primary versus relapse tumor samples, only nine shared genes identified in patient 1 may have a driver gene potential for tumor evolution. Concerning the other patients, no such shared genes were classified into Tier 3, suggesting that these genes are more likely passenger-genes, or alternatively, that the relapse is a rare de novo tumor. However, dividing driver- versus passenger-genes is challenging and should be addressed in larger cohorts or functional studies.

Genetic diversity complicates our understanding of treatment failure. Hopefully, our study will complement to the existing knowledge in the field and aid to select genes which in subsequent functional studies might prove to act as potent future biomarkers in NBL.

## Material & methods

### Ethical statement

The study protocol was approved by the Regional Committee for Medical and Health Research Ethics (REK nr: 2014/2010/REK Sør-Øst C). For all patients, written informed consents have been signed and approved by the patient or the parents of the patient, depending of the age at time of diagnosis (below/above 16 years) (REK nr: 2014/2010/REK Sør-Øst C). The parents made a voluntary and deliberate decision regarding their child’s participation in the study, based on what is best for their child, their child’s opinion, as well as their own perspectives. All methods were performed in accordance with the relevant guidelines and regulations enacted by the Genomics and Bioinformatics Core Facility, Oslo University Hospital, the South-Eastern Norway Regional Health Authority and the University of Oslo.

### Patient material

Primary and relapse tumor samples were collected for diagnostic purpose at the Department of Pathology at Oslo University Hospital Radiumhospitalet, Oslo, Norway. According to the INRG pre-treatment risk classification [9, 10] 16 patients included in the study were primarily diagnosed with high-risk NBL, one with intermediate risk, and one with very low risk disease. Patients have been treated individually but all following the HR-NBL1/SIOPEN protocol (ClinicalTrials.gov:NCT01704716) or non-HR NBL protocols when diagnosed with intermediate- or very low risk (ClinicalTrials.gov:NCT01728155) [71–75]. All high-risk cases received high dose treatment. The relapsed patients did not follow defined protocols. Blood or BM from included patients, were used as normal control material for WES analysis.

### DNA isolation

For DNA extraction different extraction kits were used, depending on the origin of the material: for fresh frozen primary tumor samples the Qiagen Allprep kit or QIAamp DNA mini kit was applied (Qiagen), for bone marrow cells the QIAamp DNA mini kit was used, and for blood the Qiagen EZ1 DNA blood kit was utilized (all Qiagen). For the majority of the samples, an additional cleaning step was applied using the Genomic DNA Clean & Concentrator TM10 (Zymo Research). DNA was quantified using the Qubit dsDNA HS Assay Kit (Invitrogen).

### Whole exome sequencing

The library preparation was performed using the Agilent SureSelect Human All Exon V5 following the default protocol and sequencing was performed on the HiSeq2500 using SBS chemistry V3 and paired end-sequencing (2x100bp).

### Data analysis

#### Variant calling

The raw reads from each sample, in FastQC format, were mapped (lane-wise) using BWA MEM to the human reference genome (build b37 with an added decoy contig, obtained from the GATK resource bundle) [76]. Sample-wise sorting and duplicate marking was performed on the initial alignments with Picard tools (http://broadinstitute.github.io/picard). GATK tools were subsequently used for two-step local realignment around indels, with matching samples (i.e., primary tumor and its corresponding normal) being processed together [77]. Each sample’s pair-end read information was checked for inconsistencies with Picard, and base-quality recalibration was performed by GATK. Somatic variant calling on the matching paired samples was done by using the intersection of MuTect and Strelka [78, 79]. Block substitutions were defined as somatic mutations at consecutive positions, where the variant allelic frequency of each was within 5% of the average allelic frequency of the two variants. GATK tools were used for computing coverage statistics based on the recalibrated alignment files. Details of the variant calling pipeline have been described elsewhere [80].

#### Variant annotation

Functional annotation of somatic variants were detected using the PCGR [81]. The detected variants were categorized into Tier 1 (*strong clinical significance*), Tier 2 (*potential clinical significance*), Tier 3 (*uncertain clinical significance*), or Tier 4 (other *non-synonymous*). The VCF files from the variant calling pipeline were compressed and indexed using bgzip and tabix, respectively, as recommended by PCGR. The PCGR script, pcgr.toml, was modified to turn off VCF validation and configured with specific parameters (Peripheral_Nervous_System_Cancer_NOS) for this analysis, as NBL falls inside this main category. The PCGR provided a list of variants in each of our 18 patients. In the PCGR, every somatic variant presented to the program is classified either as Tier 1, Tier 2, Tier 3, Tier 4, or synonymous variant. Tier 1 variants are variants known to be of *strong clinical significance* for the cancer type specified by the user, in our case NBL. Tier 2 variants are described as variants with *potential clinical significance*: either strong evidence that the variant has a clinical significance in another cancer type or weak evidence that the variant has clinical significance in the cancer type specified by the user. Tier 1 and Tier 2 variants have to be classified in the CIViC database or the Cancer Biomarkers Database [82, 83]. Tier 3 variants are variants of *uncertain clinical significance*, which are unspecified non-synonymous variants located within a known tumor suppressor gene or proto-oncogene. All other non-synonymous variants are classified into Tier 4.

The PCGR uses mutation effect predictors to estimate the biological effect of the non-synonymous variants. In this study, the mutation effect predictor MutationAssessor was chosen to identify biologically relevant variants [39]. When MutationAssessor predicts a variant with damaging effect, the amino acid change caused by that variant is predicted to cause damage to the protein product that impacts the function of the protein. If the variant is predicted to be tolerated, this means that the resulting amino acid change in the protein is predicted to have no functional impact. In this study, we use this definition for a variant to be: predicted to be damaging or predicted to be tolerated. Variant annotation was performed in this study by using the MutationAssessor with high sensitivity as a variant effect predictor [84]. This choice was necessary to avoid losing data of possible interest. Therefore, there might be some false positives (variants that are not damaging) amongst the variants that we classify as biologically relevant, hence the functional importance of candidate genes and variants should be validated.

The other feature of PCGR utilized in this study is Oncoscore [85]. This is a score between 0 and 1 expressing the frequency of whether a gene has been reported in relation to cancer in the scientific literature. A low score represents a low association, while a high score represents high association. If a variant is classified into Tier 4, Oncoscore can help identify its potential as a target for further investigation.

#### Statistical analysis

Data analyses after utilizing PCGR, including frequency analysis, statistics, and plotting, were performed using the programming language R in the integrated development environment RStudio. Various statistical algorithms for analysis of sequencing data were tested and evaluated. Genes were ranked based on their average amount of mutations across patients (G), their average amount of mutations normalised by gene length (nG), and the total number of patients with mutations in the genes (B).

#### Pathway analysis

To identify pathways impacted by the identified mutated variants GOSeq analysis was performed. [86] The analysis was carried out separately for each sample and all genes with at least one somatic mutation (Tier 1—Tier 4) based on the PCGR analysis were included. We looked at mutations in the coding region. The output of this analysis was a ranking of the Gene Ontology (GO) categories according to the number of samples for which the GO category was significant (p<0.05).

## Supporting information

S1 Table The total number of variants detected in primary tumor samples of high-risk NBL patients classified into the different Tiers. (DOCX)

S2 Table The total number of variants detected in relapse tumor samples of high-risk NBL patients classified into the different Tiers. (DOCX)

S3 Table The total number of variants detected in primary tumor vs relapse samples. PT, primary tumor; RT, relapsed tumor; RT2, second relapsed tumor.
(DOCX)

S4 Table Genes with coding mutations classified into Tier 4, reported as predicted to be damaging. (DOCX)

S5 Table Functional analysis results. Functional analysis results of the genes with somatic mutations was performed using GOSeq (1). The analysis was carried out separately for 18 primary tumor and 6 relapsed samples, performed based on the coding mutations. All genes with at least one somatic mutation (Tier 1-Tier 4) detected by the PCGR analysis were included. Information about number of samples with detected pathways and number of genes involved in the certain pathway is provided.
(XLSX)

S6 Table Tumor percentage of NBL patients included in the study. (DOCX)

S7 Table Tumor allele frequency (TAF) for detected genes. TAF = "Fraction of tumor alternate allele reads on the plus strand (TAP/(TAP+TAM))"; *, relapse.
(XLSX)
