# Correction: Whole exome sequencing of high-risk neuroblastoma identifies novel non-synonymous variants

**DOI:** 10.1371/journal.pone.0306687

**Published:** 2024-07-01

**Authors:** Weronika Przybyła, Kirsti Marie Gjersvoll Paulsen, Charitra Kumar Mishra, Ståle Nygård, Solveig Engebretsen, Ellen Ruud, Gunhild Trøen, Klaus Beiske, Lars Oliver Baumbusch

In Table 6, the SCA region and protein change of the *TTN* gene in patient 1 are incorrect and the three variants that are classified by Mutation Assessor as tolerated not damaging should be removed. Please see the correct Table 6 and its caption here.

**Table 6:** Genes with novel non-synonymous variants detected in primary tumor samples, predicted to be damaging by the MutationAssessor program, found in more than one patient. SCA–structural chromosome abnormality; chromosome locations of each variant have been included; CNV–copy number variant; ampl–amplification, na–not available.

Gene name	Chromosome location	SCA region	Protein	Patient ID
***RHBG***	1q22	c.251G>A	p.Arg84His	1
		c.428T>A	p.Val143Asp	12
***SHROOM3***	4q21.1	c.905C>T	p.Ala302Val	1
		c.3160G>T	p.Val1054Leu	12
***TTN***	2q31.2	c.53807G>A	p.Arg17936His	1
		c.78674T>C	p.Ile26225Thr	12
***FLG***	1q21.3	c.1815G>T	p.Gln605His	1
		c.3176G>T	p.Arg1059Ile	5
***UTRN***	6q24.2	c.1614G>T	p.Gln538His	1
		c.1967T>G	p.Val656Gly	4
***HLA-DRB1***	6p21.32	c.654A>T	p.Arg218Ser	5
				12
***OR6C68***	12q13.2	c.416G>A	p.Cys139Tyr	7
				12
***XIRP2***	2q24.3	c.6168G>T	p.Leu2056Phe	11
		c.5402G>A	p.Arg1801His	12
***TMEM14B***	6p24.2	c.322C>T	p.Arg108Cys	17
				14

## References

[1] Przybyła W, Gjersvoll Paulsen KM, Mishra CK, Nygård S, Engebretsen S, Ruud E, et al. (2022) Whole exome sequencing of high-risk neuroblastoma identifies novel non-synonymous variants. PLoS ONE 17(8): e0273280. doi: 10.1371/journal.pone.0273280 36037157 PMC9423626

