# Supplementary material for: Whole exome sequencing of high-risk neuroblastoma identifies novel non-synonymous variants
Source: PLoS One. 2022 Aug 29;17(8):e0273280. doi: 10.1371/journal.pone.0273280 (PMC9423626; doi:10.1371/journal.pone.0273280)
Supplement: S1 Table — (DOCX) [file pone.0273280.s001.docx]

Supporting information

Whole exome sequencing of high-risk neuroblastoma identifies novel non-synonymous variants

Weronika Przybyła ^1,2 *^, Kirsti M. G. Paulsen ^1,2^, Charitra Kumar Mishra^3,4^, Ståle Nygård ^4^, Solveig Engebretsen^5^, Ellen Ruud ^2,6^, Gunhild Trøen ^7^

Klaus Beiske ^2, 7^, and Lars Oliver Baumbusch^1^

^1^Department of Pediatric Research, Division of Paediatric and Adolescent Medicine, Oslo University Hospital Rikshospitalet, Oslo, Norway

^2^Medical Faculty, Institute of Clinical Medicine, University of Oslo, Oslo, Norway

^3^Bioinformatics Core Facility, Institute for Cancer Research, Oslo University Hospital, Oslo, Norway
^4^ELIXIR-Norway, Institute of Informatics, University of Oslo, Oslo, Norway

^5^Norwegian Computing Center, Oslo, Norway

^6^Department of Paediatric Haematology and Oncology, Division of Paediatric and Adolescent Medicine, Oslo University Hospital, Rikshospitalet, Oslo, Norway

^7^Department of Pathology, Oslo University Hospital Radiumhospitalet, Oslo, Norway

*Corresponding author:

E-mail: [weronika.przybyla@studmed.uio.no](mailto:weronika.przybyla@studmed.uio.no) (WP)

**S1 Table.** The total number of variants detected in primary tumor samples of high-risk NBL patients classified into the different Tiers.

| **Patient ID** | **Total # variants** | **Tier 1** | **Tier 2** | **Tier 3** | **Tier 4** | **Synonymous** |
| --- | --- | --- | --- | --- | --- | --- |
| 1 | 770 | - | - | 29 | 262 | 479 |
| 2 | 12 | - | - | - | 6 | 6 |
| 4 | 49 | - | - | 3 | 13 | 33 |
| 5 | 129 | - | - | 3 | 51 | 75 |
| 6 | 96 | - | - | - | 31 | 65 |
| 7 | 185 | - | - | 4 | 40 | 141 |
| 8 | 12 | - | - | - | 3 | 9 |
| 9 | 39 | - | - | 2 | 3 | 34 |
| 11 | 87 | - | - | 5 | 32 | 50 |
| 12 | 1687 | - | 2 | 21 | 346 | 1318 |
| 13 | 21 | - | - | 1 | 7 | 13 |
| 14 | 19 | - | - | 1 | 7 | 11 |
| 15 | 24 | - | - | - | 7 | 17 |
| 16 | 55 | - | 1 | 1 | 15 | 38 |
| 17 | 63 | - | - | 1 | 22 | 40 |
| 18 | 104 | - | - | 3 | 35 | 66 |
| 20 | 53 | - | - | 3 | 22 | 28 |
| 22 | 21 | - | 1 | - | 4 | 16 |
