# Supplementary material for: Whole exome sequencing of high-risk neuroblastoma identifies novel non-synonymous variants
Source: PLoS One. 2022 Aug 29;17(8):e0273280. doi: 10.1371/journal.pone.0273280 (PMC9423626; doi:10.1371/journal.pone.0273280)
Supplement: S3 Table — PT, primary tumor; RT, relapsed tumor; RT2, second relapsed tumor. (DOCX) [file pone.0273280.s003.docx]

Supporting information

Whole exome sequencing of high-risk neuroblastoma identifies novel non-synonymous variants

Weronika Przybyła ^1,2 *^, Kirsti M. G. Paulsen ^1,2^, Charitra Kumar Mishra^3,4^, Ståle Nygård ^4^, Solveig Engebretsen^5^, Ellen Ruud ^2,6^, Gunhild Trøen ^7^

Klaus Beiske ^2, 7^, and Lars Oliver Baumbusch^1^

^1^Department of Pediatric Research, Division of Paediatric and Adolescent Medicine, Oslo University Hospital Rikshospitalet, Oslo, Norway

^2^Medical Faculty, Institute of Clinical Medicine, University of Oslo, Oslo, Norway

^3^Bioinformatics Core Facility, Institute for Cancer Research, Oslo University Hospital, Oslo, Norway
^4^ELIXIR-Norway, Institute of Informatics, University of Oslo, Oslo, Norway

^5^Norwegian Computing Center, Oslo, Norway

^6^Department of Paediatric Haematology and Oncology, Division of Paediatric and Adolescent Medicine, Oslo University Hospital, Rikshospitalet, Oslo, Norway

^7^Department of Pathology, Oslo University Hospital Radiumhospitalet, Oslo, Norway

*Corresponding author:

E-mail: [weronika.przybyla@studmed.uio.no](mailto:weronika.przybyla@studmed.uio.no) (WP)

**S3 Table.** The total number of variants detected in primary tumor vs relapse samples. PT, primary tumor; RT, relapsed tumor; RT2, second relapsed tumor.

| **Patient ID** | **Total nr of variants** | | |
| --- | --- | --- | --- |
|  | **PT** | **RT** | **RT2** |
| 1 | 770 | 1229 | - |
| 6 | 96 | 260 | - |
| 7 | 185 | 252 | 274 |
| 17 | 63 | 493 | - |
| 23 | na | 199 | - |
