# Supplementary material for: Whole exome sequencing of high-risk neuroblastoma identifies novel non-synonymous variants
Source: PLoS One. 2022 Aug 29;17(8):e0273280. doi: 10.1371/journal.pone.0273280 (PMC9423626; doi:10.1371/journal.pone.0273280)
Supplement: S4 Table — (DOCX) [file pone.0273280.s004.docx]

Supporting information

Whole exome sequencing of high-risk neuroblastoma identifies novel non-synonymous variants

Weronika Przybyła ^1,2 *^, Kirsti M. G. Paulsen ^1,2^, Charitra Kumar Mishra^3,4^, Ståle Nygård ^4^, Solveig Engebretsen^5^, Ellen Ruud ^2,6^, Gunhild Trøen ^7^

Klaus Beiske ^2, 7^, and Lars Oliver Baumbusch^1^

^1^Department of Pediatric Research, Division of Paediatric and Adolescent Medicine, Oslo University Hospital Rikshospitalet, Oslo, Norway

^2^Medical Faculty, Institute of Clinical Medicine, University of Oslo, Oslo, Norway

^3^Bioinformatics Core Facility, Institute for Cancer Research, Oslo University Hospital, Oslo, Norway
^4^ELIXIR-Norway, Institute of Informatics, University of Oslo, Oslo, Norway

^5^Norwegian Computing Center, Oslo, Norway

^6^Department of Paediatric Haematology and Oncology, Division of Paediatric and Adolescent Medicine, Oslo University Hospital, Rikshospitalet, Oslo, Norway

^7^Department of Pathology, Oslo University Hospital Radiumhospitalet, Oslo, Norway

*Corresponding author:

E-mail: [weronika.przybyla@studmed.uio.no](mailto:weronika.przybyla@studmed.uio.no) (WP)

**S4 Table.** Genes with coding mutations classified into Tier 4, reported as predicted to be damaging.

| **Patient ID** | **Gene name** | **Oncoscore** | **Protein Change** |
| --- | --- | --- | --- |
| 2 | *NCKAP5* | ~0.26 | p.Gly687Trp |
|  |  |  | c.2059G>T |
| 6 | *TNIK* | ~0.62 | p.Pro689His |
|  |  |  | c.2066C>A |
| 8 | *PSMC3* | ~0.27 | p.Arg304Trp |
|  |  |  | c.910C>T |
| 15 | *AHNAK2* | ~0.60 | p.Lys3142Arg |
|  |  |  | c.9425A>G |
