# Supplementary material for: Whole exome sequencing of high-risk neuroblastoma identifies novel non-synonymous variants
Source: PLoS One. 2022 Aug 29;17(8):e0273280. doi: 10.1371/journal.pone.0273280 (PMC9423626; doi:10.1371/journal.pone.0273280)
Supplement: S6 Table — (DOCX) [file pone.0273280.s006.docx]

Supporting information

Whole exome sequencing of high-risk neuroblastoma identifies novel non-synonymous variants

Weronika Przybyła^1,2 *^, Kirsti M. G. Paulsen^1,2^, Charitra Kumar Mishra^3,4^, Ståle Nygård^4^, Solveig Engebretsen^5^, Ellen Ruud^2,6^, Gunhild Trøen^7^

Klaus Beiske^2, 7^, and Lars Oliver Baumbusch^1^

^1^Department of Pediatric Research, Division of Paediatric and Adolescent Medicine, Oslo University Hospital Rikshospitalet, Oslo, Norway

^2^Medical Faculty, Institute of Clinical Medicine, University of Oslo, Oslo, Norway

^3^Bioinformatics Core Facility, Institute for Cancer Research, Oslo University Hospital, Oslo, Norway
^4^ELIXIR-Norway, Institute of Informatics, University of Oslo, Oslo, Norway

^5^Norwegian Computing Center, Oslo, Norway

^6^Department of Paediatric Haematology and Oncology, Division of Paediatric and Adolescent Medicine, Oslo University Hospital, Rikshospitalet, Oslo, Norway

^7^Department of Pathology, Oslo University Hospital Radiumhospitalet, Oslo, Norway

*Corresponding author:

E-mail: [weronika.przybyla@studmed.uio.no](mailto:weronika.przybyla@studmed.uio.no) (WP)

**S6 Table. Tumor percentage of NBL patients included in the study.**

| **Patient ID** | **Tumor percentage [%]** |
| --- | --- |
| 1 | 90 |
| 1 * | 80 |
| 2 | 85 |
| 4 | 70 |
| 5 | 75 |
| 6 | 70 |
| 6 * | 80 |
| 7 | 80 |
| 7 * | 75 |
| 7 ** | 80 |
| 8 | 60 |
| 9 | 70 |
| 11 | 85 |
| 12 | 85 |
| 13 | na |
| 14 | 60 |
| 15 | 90 |
| 16 | 70 |
| 17 | 85 |
| 17 * | 70 |
| 18 | 80 |
| 19 | 80 |
| 20 | 90 |
| 21 | 85 |
| 22 | 90 |
| 23 | 85 |

Amount of tumor cells identified in each sample at time of diagnosis and relapse. Samples originating from two relapses of patient 7 are available: 7* and 7**; *, relapse.
